# Pre-operative lung ablation prediction using deep learning

**DOI:** 10.1007/s00330-024-10767-8

**Published:** 2024-05-22

**Authors:** Krishna Nand Keshavamurthy, Carsten Eickhoff, Etay Ziv

**Affiliations:** 1 Memorial Sloan Kettering Cancer Center, 1275 York Avenue, New York, NY 10065 USA; 2 University of Tübingen Geschwister-Scholl-Platz, 72074 Tübingen, Germany

**Keywords:** Microwave ablation, Patient specific modeling, Treatment prediction, Data driven, Deep learning

## Abstract

**Objective:**

Microwave lung ablation (MWA) is a minimally invasive and inexpensive alternative cancer treatment for patients who are not candidates for surgery/radiotherapy. However, a major challenge for MWA is its relatively high tumor recurrence rates, due to incomplete treatment as a result of inaccurate planning. We introduce a patient-specific, deep-learning model to accurately predict post-treatment ablation zones to aid planning and enable effective treatments.

**Materials and methods:**

Our IRB-approved retrospective study consisted of ablations with a single applicator/burn/vendor between 01/2015 and 01/2019. The input data included pre-procedure computerized tomography (CT), ablation power/time, and applicator position. The ground truth ablation zone was segmented from follow-up CT post-treatment. Novel deformable image registration optimized for ablation scans and an applicator-centric co-ordinate system for data analysis were applied. Our prediction model was based on the U-net architecture. The registrations were evaluated using target registration error (TRE) and predictions using Bland-Altman plots, Dice co-efficient, precision, and recall, compared against the applicator vendor’s estimates.

**Results:**

The data included 113 unique ablations from 72 patients (median age 57, interquartile range (IQR) (49–67); 41 women). We obtained a TRE ≤ 2 mm on 52 ablations. Our prediction had no bias from ground truth ablation volumes (*p* = 0.169) unlike the vendor’s estimate (*p* < 0.001) and had smaller limits of agreement (*p* < 0.001). An 11% improvement was achieved in the Dice score. The ability to account for patient-specific in-vivo anatomical effects due to vessels, chest wall, heart, lung boundaries, and fissures was shown.

**Conclusions:**

We demonstrated a patient-specific deep-learning model to predict the ablation treatment effect prior to the procedure, with the potential for improved planning, achieving complete treatments, and reduce tumor recurrence.

**Clinical relevance statement:**

Our method addresses the current lack of reliable tools to estimate ablation extents, required for ensuring successful ablation treatments. The potential clinical implications include improved treatment planning, ensuring complete treatments, and reducing tumor recurrence.

## Key Points

- *Reliable tools to predict the extent of ablation treatments are currently lacking.*
- *Our novel patient-specific deep-learning algorithm was shown to predict ablation zones with higher accuracy and less bias compared to the currently used estimates provided by applicator vendor.*
- *Our method for ablation prediction allows for real-time clinical deployment, with potential for improved treatment planning and reduced tumor recurrence.*

## Introduction

Microwave lung ablation (MWA) is used to eradicate tumors in the lung [1], and offers a low morbidity and inexpensive alternative to surgical resection [2]. Additionally, MWA shows no detectable long-term effects on pulmonary function, making it an attractive alternative to stereotactic body radiation therapy [3]. However, widespread adoption of MWA has been hindered by higher local recurrence rates [4], which result from incomplete ablations with insufficient tumor margins [5].

Achieving adequate margins (> 5 mm) with MWA is challenging [5]. The ablation device vendors provide a chart of the expected ablation/treatment zone dimensions as a function of power and duration (henceforth referred to as the *vendor model*), which is used for pre-procedure planning. The vendor model is based on ablations of ex-vivo animal organs. However, clinical ablation zones are affected by nearby vessels and airways that act as heat sinks, pleural boundaries that cause distortions, and complex lung deformations due to patient position and breathing. Observed ablation zones demonstrate marked deviation from the vendor models in size and shape [6–8]. There is wide variability in ablation zones even for the same power and duration settings [6, 9, 10]. As a result, the treatment plan based on the vendor model may significantly differ from the actual treatment, potentially leading to incomplete thermal destruction of tumors and failure to establish an adequate margin.

Biophysical models that attempt to simulate ablation zones by solving partial differential equations governing the physical processes during MWA [11, 12] are limited in their ability to account for patient-specific tissue properties, lack clinical validation, and are too computationally demanding for clinical application. In a recent study relating “antenna work” (power x duration) with ablation zone volume, the authors noted the wide range of volumes for similar work and emphasized the importance of accounting for patient-specific characteristics [10].

In this work, we introduce a new, data-driven approach for ablation prediction. We hypothesize that pre-procedure computerized tomography (CT) scan intensity features contain patient-specific characteristics that can inform the ablation zone size and shape, and along with the ablation settings, are predictive of the final ablation zone extents. We treat the problem as that of learning a deep-neural network-based parameterized function that takes the pre-procedure CT, the position of the applicator, and ablation power and duration as input and predicts the post-procedure ablation zone as output. Our goal is to predict the final ablation zone at the first follow-up post-procedure scan.

## Materials and methods

### Data

**Fig. 1:**
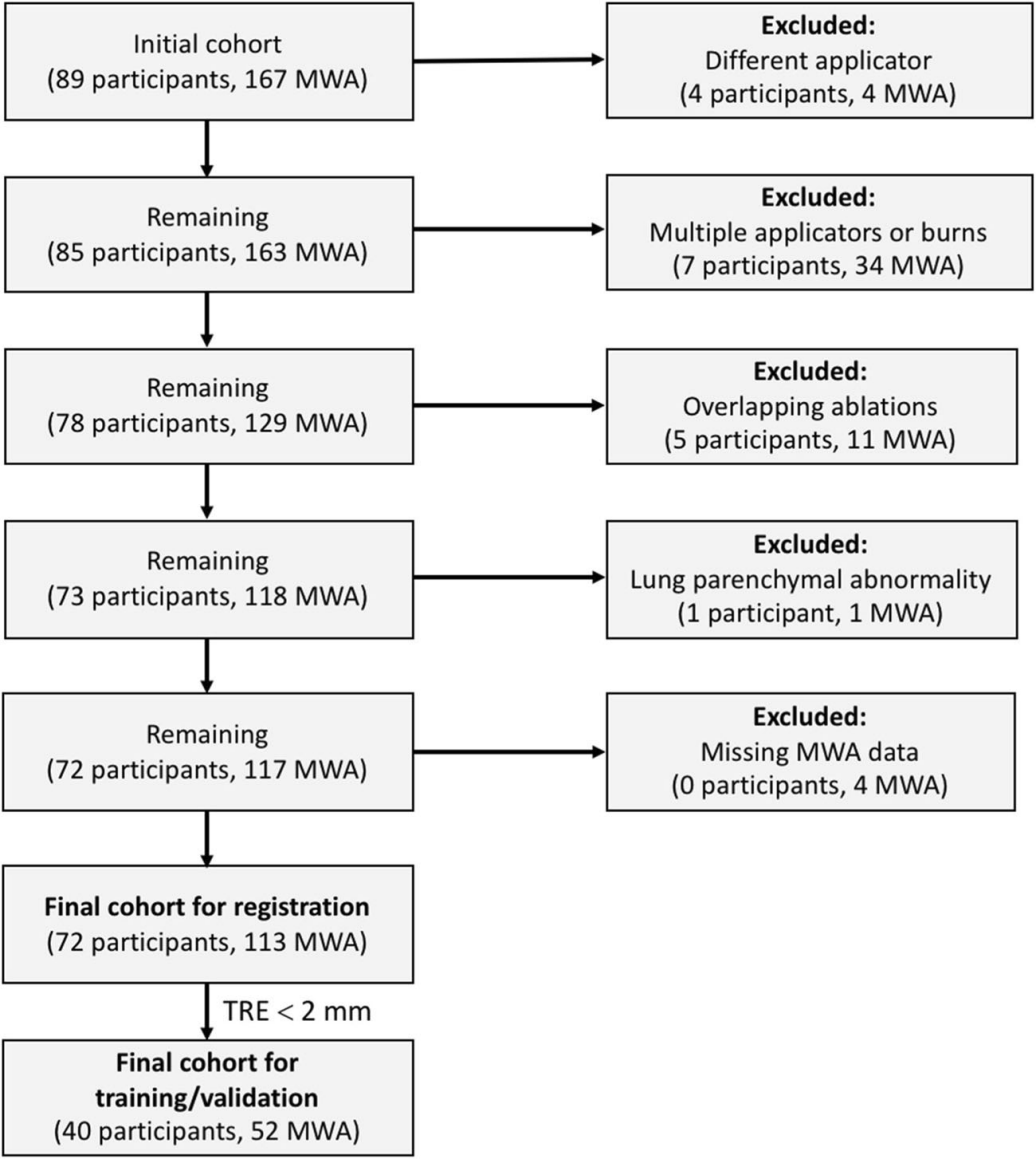
Flow diagram of number of participants and number excluded. Lung microwave ablation (MWA) and target registration error (TRE). Final cohort for registration: 72 participants, 113 ablations; median IQR age: 57 (49–67); gender: 41 women, 31 men. Final cohort for training/validation (TRE ≤ 2 mm): 40 participants, 52 ablations; median IQR age: 56 (49–65); gender: 20 women, 20 men

The data was obtained as part of an institutional review board approved (IRB/Privacy Board-A, protocol 17-353)^1^ retrospective study of patients who underwent MWA at our institution between 01/2015 and 01/2019. Exclusion criteria included the use of a probe other than NeuWave PR (Ethicon US), multiple probes or multiple burns performed at the same site, two or more adjacent sites with overlapping ablation zones, or if background lung parenchyma could not be differentiated from the ablation zone, resulting in 113 unique ablations from 72 patients (see Fig. 1). Pre-procedure and 1-month follow-up post-procedure CT scans were processed. MWA power and duration were obtained from radiology reports.

### Pre-processing pipeline

**Fig. 2:**
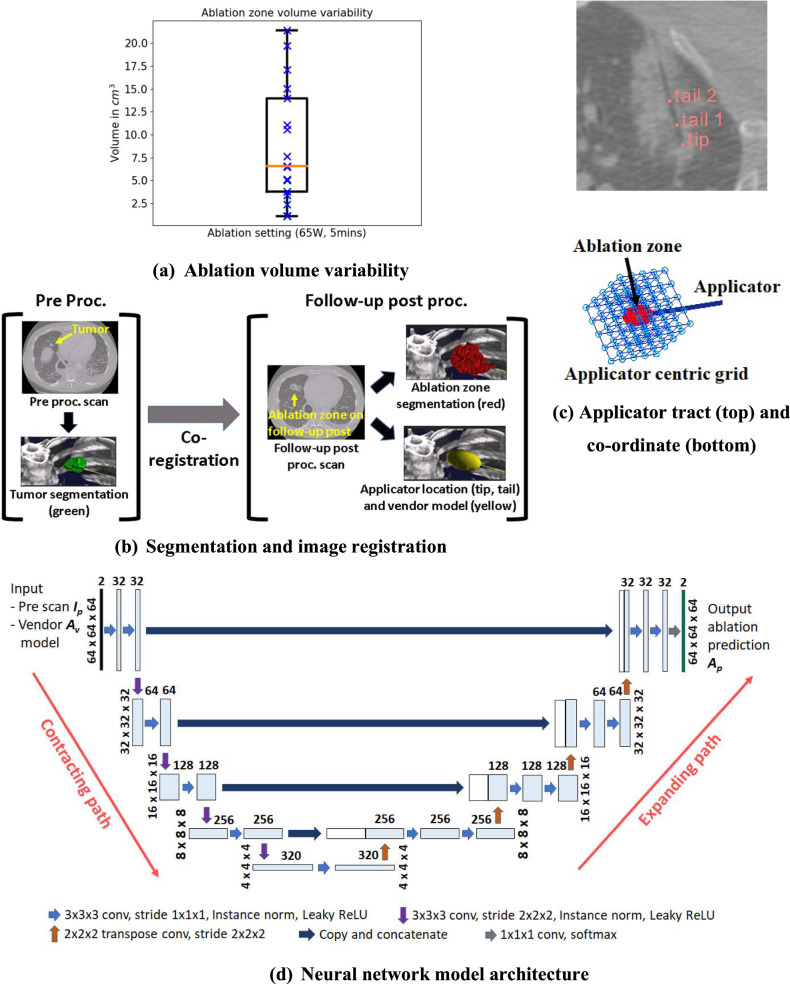
**a** Ablation zone volume variability for the same power (65 W) and duration (5 min) of ablation. **b** Segmentation of tumor in pre and ablation zones in follow-up post scans, and deformable image registration between pre and post. **c** Applicator tract (tip/tails) on follow-up post scan (top) and applicator-centric co-ordinate system for all data analysis (bottom). **d** Our neural network model

Figure 2b, c provide an overview of the pre-processing steps in our pipeline. The tumor and ablation zone were manually segmented in the pre and follow-up scans, respectively (Fig. 2b), by a radiologist with > 15 years of experience [13]. Contours were drawn marking the boundary of the tumor and ablation zone on axial CT image slices in 3D Slicer software (v4.11.1) [14], and further edited on coronal and sagittal views. As ablation zones can have internal regions of high or low Hounsfield units (HU), all pixels within the drawn boundary were assumed to be part of the segmentation independent of the HU. The applicator position was defined by the radio-lucent tract visible in the ablation zone on the follow-up scan (Fig. 2c).

For the vendor model, we used the vendor’s user interface software (v3.1.0), which provided dimensions of expected ablation ellipsoids for different powers (35 W to 65 W in 5 W increments) and durations (1 to 10 min in 1 min increments). Ablation zone dimensions were linearly interpolated for power and duration in between these specifications.

We applied a novel deformable image registration methodology for ablation procedures, utilizing the free-form deformation model based on B-spline transformation [15] to register the pre and follow-up scans (Fig. 2b). An initial rigid registration was performed for coarse alignment followed by a coarse-to-fine multi-resolution deformable registration where the result of each resolution acted as input to the next [16]. We constrained the registration by focusing on the regions surrounding the tumor and ablation zone and applied a rigidity penalty on the tumor to avoid large tumor deformations. The registration optimization used mutual information to measure the similarity between images and restricted excessive bending of the transform to avoid unnatural deformations via regularization. We followed Klein et al to set various registration parameters [17]. More details on the registration methodology can be found in Supplemental G. All registrations were performed using the SimpleElastix image registration library (v0.9.1) [18, 19].

To enable comparison of ablation zones at different positions/orientations across patients, we defined an applicator-centric co-ordinate system (ACCS) [20], an oblique 3D co-ordinate grid oriented along the applicator and centered at the applicator tip (Fig. 2c) for all computation. The ACCS grid size was set to 64 × 64 × 64 mm^3^ with a sampling rate of 1 mm along each dimension. Numpy (v1.21.2) [21], SciPy (v1.7.3) [22], and NiBabel (v3.2.1) [23] Python libraries were used for data processing.

### Model

Our model was a fully convolutional neural network based on the U-Net [24] architecture (Fig. 2d and Supplemental A). The U-net consists of (1) the encoder that reduces the dimensionality of the input, aggregating semantic information (*what is in the image*), (2) the decoder that combines the encoded semantic information with higher resolution spatial information from initial encoding layers via skip connections (*where in the image*) and up-samples them to the original size to produce output. The first input channel was the 3D pre-CT scan. The ablation power/duration is represented by the dimensions of the vendor ellipsoid, which was hence used as the 2nd input channel after placing at the applicator position.

### Training

The segmented ablation zones on follow-up scans were used as ground truth for training since they are the clinical standard for assessing lung ablation zones and treatment margins [25, 26]. We utilized the sum of cross entropy and the Dice co-efficient between the predicted and ground truth as the loss function to train the network [27]. We performed nested cross-validation [28] for training and testing (Supplemental B): (1) In the outer loop, we performed 7-fold cross-validation (CV) [29] resulting in 7 *train-test (TT)* CV splits, where, in each split, 6 folds were used for model training and the remaining 7th fold (unseen during training) used for testing. The results on the test folds from all the 7 TT splits were averaged to compute the final test set performance [29, 30]. (2) In the inner loop, the training data in each TT split (6 folds) was further split into 5 *train-validation (TV)* CV splits, where, in each TV split, the model was trained on 4 folds and tuned on the remaining 5th validation fold held out during training. The purpose of these further CV splits was to train different models on each of the 5 TV splits of the training data from a single TT split, which were then applied on the corresponding TT split test fold and the results were ensembled by averaging their softmax outputs. Hence, we trained 5 different models from the training data of each TT split, resulting in a total of 7 × 5 = 35 models on the whole dataset. We followed Isensee et al for the training methodology (Supplemental B; https://github.com/MIC-DKFZ/nnUNet) [27, 31]. The code for pre-processing and model training can be accessed at https://github.com/tenres/Abl-Pred.

### Statistics

Image registrations were evaluated using the target registration error (TRE; Supplemental C) [32]. Volumes and shapes of our prediction and vendor model were compared with ground truth ablation zones using Bland-Altman plots [33]. Paired and two sample *t*-tests were used to test for differences between the ground truth and vendor model. Levene’s test was used to test the differences in the limits of agreement (LOA) [34]. The degree of overlap between prediction and ground truth was computed using the Dice co-efficient [35], precision, and recall [36] (Supplemental D). The mean and 95% confidence intervals (CIs) were computed [29], which were compared against that of the vendor model. Statsmodel (v0.13.5) [37], and SciPy (v1.7.3) [22] Python libraries were used for all analyses.

## Results

After exclusions, there were 72 participants (median age 57 years, 47–69 (interquartile range, IQR); 41 women) with 113 MWA procedures (Fig. 1). Cases with poor TRE (> 2 mm) were excluded from training/testing, resulting in 52 ablations from 40 patients (Fig. 1). Table 1 lists tumor, cancer, and CT imaging details. There were 17 ablations performed using the median power of 65 watts (range 20–65 watts), and duration of 5 min (range 1–10 min), and the variability in their ablation zone volumes (same power/duration) is shown in Fig. 2a. The TRE on the final 52 cases is shown in Fig. 4a with a median of 0.88, IQR (0.50–1.31). The inference time of our algorithm was a fraction of a second.

**Table 1:** Data characteristics

Data characteristic	Value
Tumor largest diameter	Median = 9.0 mm; IQR = (7.0–10.2) mm
Tumor location (lung lobe, close to vs. away from pleura and close to vs. away from large vessels (> 3 mm diameter). Closeness is defined as within 5 mm).	Lung lobe: LUL = 35%, RUL = 19%, LLL = 21%, RLL = 13%, RML = 12%; close to vs. away from pleura = 33% vs. 67%; close to vs. away from large vessels = 38% vs. 62%.
Cancer diagnosis	Colon = 80%, Lung = 8%, Uterine LMS = 4%, Liver = 2%, Head and neck = 2%, Esophagus = 2%, Pancreas = 2%.
Tumor type	METS = 94%, Primary = 6%
Duration between pre/post imaging acquisition and ablation.	Pre median: 35 days; IQR = (23–48) Post median: 30 days; IQR = (24–35)
Pre/post CT median imaging parameters	Pre: CT kVp = 120; tube current = 218 mA (IQR:150.75, 294.25); slice thickness = 1.25 mm; x,y pixel spacing = 0.69 mm (IQR: 0.65–0.74) Post: CT kVp = 120; tube current = 239.5 mA (IQR:170.0, 295.5); slice thickness = 1.25 mm; x,y pixel spacing = 0.68 mm (IQR: 0.62–0.73)

*LUL* left upper lobe, *LLL* left lower lobe, *RUL* right upper lobe, *RLL* right lower lobe, *RML* right middle lobe, *METS* metastatic disease, *kVp* kilovoltage peak, *IQR* interquartile range

### Bias and variability of volume and shape for our prediction and vendor models

**Fig. 3:**
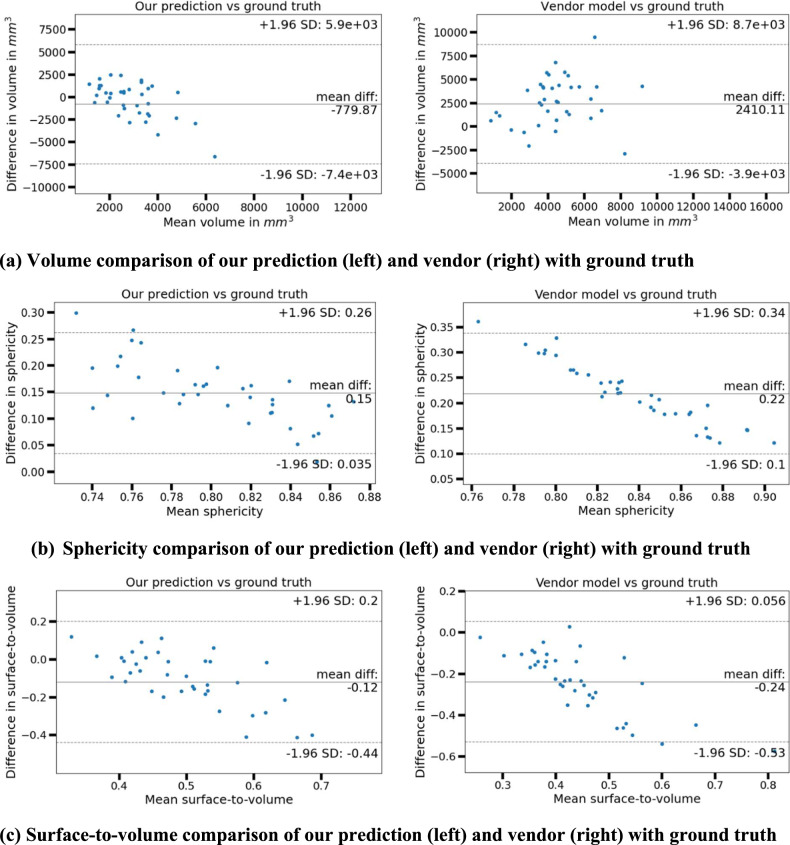
Bland-Altman plots comparing the volumes and shapes of our prediction and vendor model with ground truth. A comparison of volumes is shown in **a**, sphericity in **b**, and surface-to-volume ratio in **c**. The bias (mean difference) is shown by the solid line and the limits of agreement (1.96 SD of the difference on either side of the bias) are shown by dotted lines in each case. Note the range of values along the axes is different for different plots to ensure optimal visualization at different scales

Bland-Altman plots comparing the volume and shape characteristics of our prediction and vendor model against the true ablation zone are shown in Fig. 3. The volume bias (Fig. 3a) of our prediction (–780), was lower compared to the vendor model (2410) and this difference was significant (*p* < 0.001). Assuming differences from ground truth to be normally distributed (*y*-axis), the bias of our prediction was not different from the line of equality with ground truth (0 bias) (*p* = 0.169); whereas, the vendor model bias was significant, *p* < 0.001. The range in the LOA of our model was smaller than the vendor model and this difference was significant (*p* < 0.001). The sphericity (Fig. 3b) and surface-to-volume ratio (Fig. 3c) biases of our prediction were lower compared to the vendor model (0.15 vs. 0.22 and –0.12 vs. –0.24, *p* < 0.001). The volume and shape differences demonstrate better agreement of our prediction with the true ablation zone compared to vendor.

### Dice score, precision, recall

**Fig. 4:**
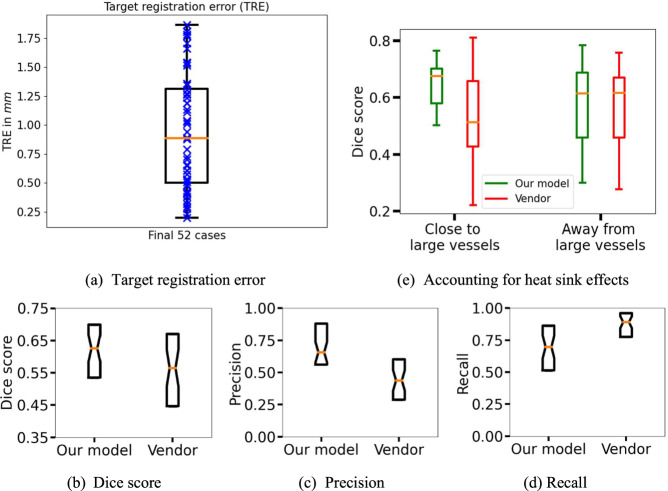
Boxplots of various performance metrics, where each box extends from the lower to the upper quartile values with the median shown in orange. Reading counterclockwise: **a** Target registration error for the final 52 cases. **b** Dice scores, **c** precision, and **d** recall quantify the overlap between prediction and ground truth. Notches around the median represent the 95% confidence interval. The median Dice score, precision, and recall were 0.62, 0.65, and 0.70 for our model compared with 0.56, 0.43, and 0.89 for the vendor model. **e** Quantitative demonstration of the ability of our model and the inability of the vendor model to account for heat-sink effects. The boxplots on the left show Dice overlap with ground truth for ablations close (within 5 mm) to large vessels (> 3 mm diameter), and those on the right show Dice for ablations away from large vessels. Our model’s Dice are shown in green and vendor model’s in red

We calculated the Dice scores of our prediction and vendor model with the true ablation zones (Fig. 4b and Table 2). The median Dice scores on the test set were 0.62 ± 0.12 (CI: 0.56–0.64) for our model compared with 0.56 ± 0.16 (CI: 0.49–0.59) for the vendor model, demonstrating an 11% improvement over the vendor model. The vendor model also showed a broader IQR consistent with the large LOA range. We saw improved precision of our model with median scores of 0.65 ± 0.22 (CI: 0.60–0.75) compared with 0.43 ± 0.23 (CI: 0.39–0.54) for the vendor model (Fig. 4c and Table 2). The vendor model demonstrated better recall with median scores of 0.89 ± 0.14 (CI: 0.79–0.89) compared with 0.70 ± 0.22 (CI: 0.60–0.73) for our model.

**Table 2:** Dice scores, precision, and recall for our model and the vendor model

Method	Dice	Precision	Recall
**Ours**	**0.60** ± **0.12 (0.56–0.64)**	**0.67** ± **0.22 (0.60–0.75)**	0.66 ± 0.22 (0.60–0.73)
**Vendor**	0.54 ± 0.16 (0.49–0.59)	0.46 ± 0.23 (0.39–0.54)	**0.84** ± **0.14 (0.79–0.89)**

In each cell, the top row shows the mean and standard deviation and the bottom row shows the 95% confidence interval. The method with the higher score in each column is shown in boldface

### Effect of patient-specific local anatomy

To better understand the quantitative improvement in Dice score, we evaluated our prediction and vendor model in their ability to account for patient-specific local anatomy. Several scenarios with two examples each are presented.

#### Heat-sink effects

**Fig. 5:**
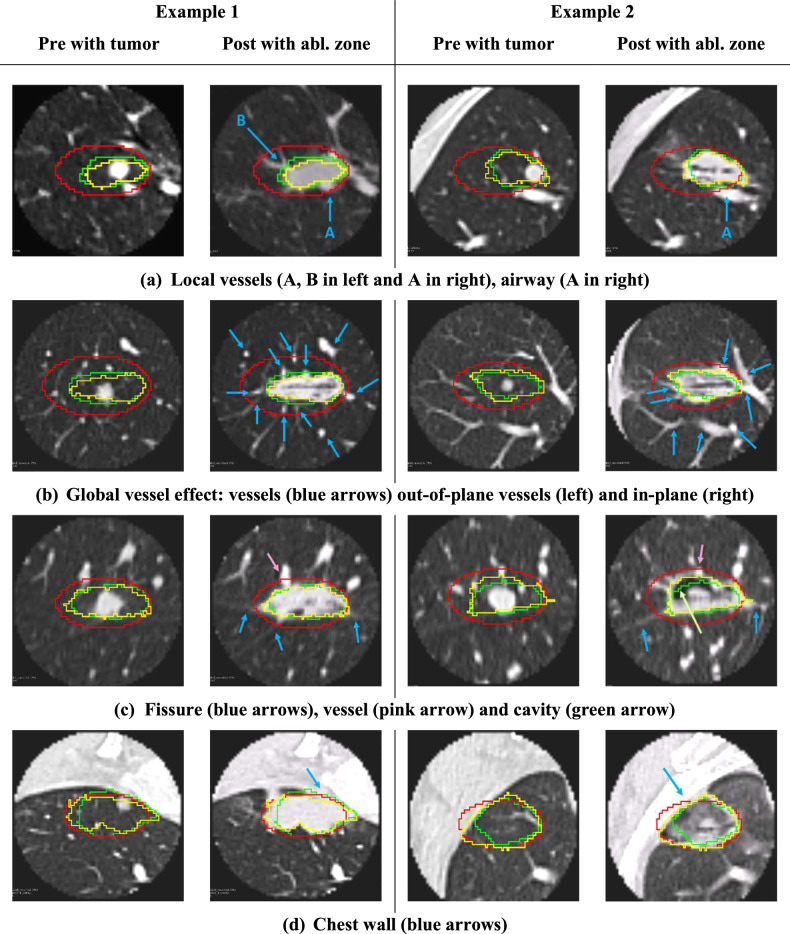
**a**–**d** demonstrate predicted ablation zones in various anatomical scenarios with 2 examples of each (left and right of midline). Our prediction (green), vendor model (red), and true ablation (yellow) are overlaid on both the pre-scan with tumor (left) and the follow-up post scan with ablation zone (right) in each example

Figure 5a demonstrates local heat-sink effects from adjacent vessels and airways that locally modulate the shape of the ablation zone. Our prediction does not extend into the vessels or airways, similar to the ground truth. In contrast, the vendor model overestimates the ablation zone and extends into the vessels as well as the background lung parenchyma. Figure 5b demonstrates global heat-sink effects on ablation zone size and shape. In example 1 (left), multiple small and medium-sized vessels (blue arrows) are seen in the cross-section coming in and out of the plane with a cumulative effect of decreasing the overall ablation zone size. Our model closely follows the true ablation zone whereas the vendor overestimates the back and sides of the applicator. Notably, the effects of vessels are not distributed symmetrically around the needle. In example 2 (right), multiple vessels (blue arrows) are now seen in the plane. Again, our prediction conforms to the asymmetric narrowing and decreased size of the ablation zone. Figure 4e demonstrates this effect quantitatively, where our model has a higher Dice overlap with true ablation compared to vendor when close to large vessels that cause heat-sink effects, whereas is similar to vendor when away from large vessels.

#### Borders

**Fig. 6:**
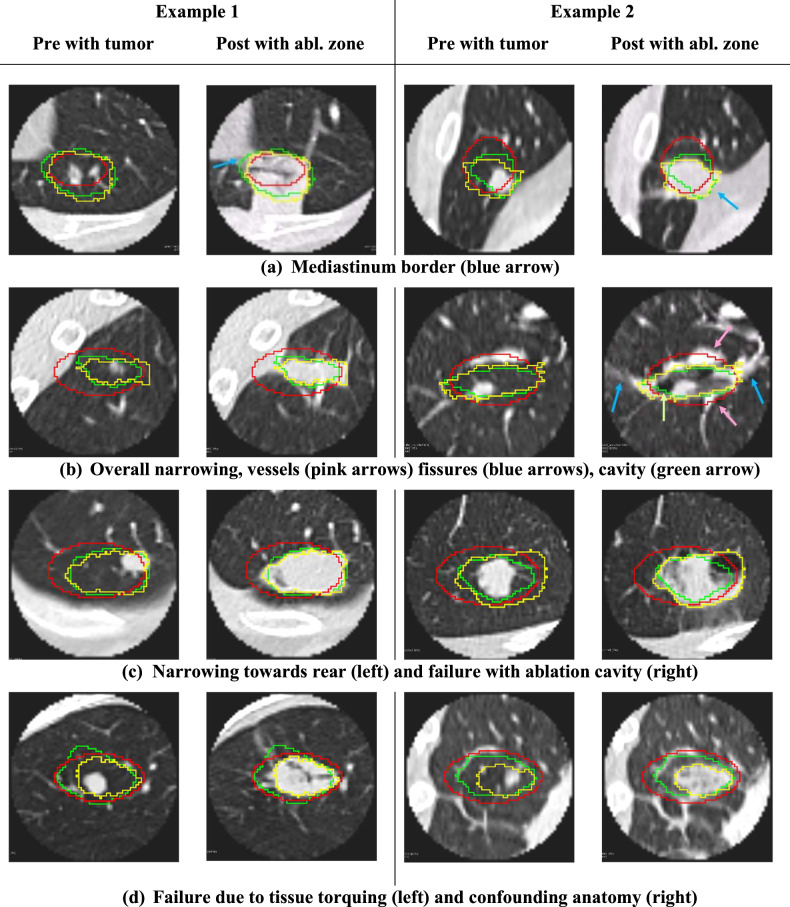
**a**–**d** demonstrate additional challenging anatomical scenarios for ablation prediction with 2 examples of each on either side of the midline. Our prediction (green), vendor model (red), and true ablation (yellow) are overlaid on both the pre-scan with tumor (left) and the follow-up post-scan with ablation zone (right) in each example. Some failure cases of our method are also shown in **c** (right example) and **d**

Figure 5c demonstrates the effects of fissures, and faint radio-dense lines demarcated by blue arrows on post images. Our prediction follows the fissure, whereas the vendor model extends beyond. Figure 5d shows the effect of chest-wall boundaries, which our prediction adheres to closely, whereas the vendor model extends beyond the boundary in example 2. Figure 6a shows mediastinal borders where our prediction adheres to the local border whereas the vendor underestimates (example 1) or overestimates (example 2) the ablation zone.

#### Ablation shape

The overall shape of the ablation zone is affected by lung anatomy. Figure 6b example 1 shows asymmetric narrowing of the ablation zone to which our model adheres closely but which the vendor model overestimates. In example 2, the ablation zone appears rotated relative to the vendor model, possibly due to the adjacent vascular structures, which our model is able to predict. Figure 6c example 1 shows our prediction narrowing only at the rear end of the ablation, similar to ground truth observation. More examples in 3D are provided in Supplemental H.

#### Failures

We also identified categories of scenarios in which our model consistently failed to predict the ablation zone. Figure 6c example 2 and Fig. 5c example 2 (green arrow) show cases where the ablation zone contains a large cavity, which our model excludes. In Fig. 6d example 1, our prediction extends too far and wide along the back of the probe. A close review of intra-procedure ablation images revealed that the lung was torqued and compressed intra-operatively during applicator positioning. Example 2 shows confounding background anatomy where our model incorrectly extends beyond the ablation zone (towards the left), adhering to scars that have an ablation border-like appearance.

## Discussion

We have presented a data-driven deep-learning model to predict lung ablation zones as they appear on the follow-up scan based on pre-procedure imaging and ablation parameters. Compared with existing vendor models, we demonstrated higher Dice scores, decreased volume variability, and decreased bias in volume and shape from true ablation. Our model is also able to better account for the effects of patient-specific local anatomy on the ablation zone including heat-sink effects, organ/lobes boundaries, and variations in the shape of the ablation zone. Moreover, we note that the improvement in Dice’s performance is likely an underestimate (see Supplemental E).

Clinically, the tradeoff between precision and recall is significant. A bias towards over-estimating the ablation zone increases recall but may cause the operator to under-treat the tumor, leading to local recurrence. Whereas a bias towards under-estimating the ablation zone can increase precision but may result in overly aggressive treatment and potential complications (Supplemental F). The low precision and high recall of the vendor model show a striking mismatch that translates clinically to over-estimation of the ablation zone, incomplete treatment, and high local recurrence. Our model showed no bias, a marked improvement in precision at the cost of some decrease in recall, potentially averting tumor under-treatment, local recurrence, and repeat procedures. Depending on location and proximity to critical structures, one could tune the threshold to modulate the precision-recall tradeoff.

Our approach offers several advantages over biophysical ablation modeling. We directly incorporate patient-specific observed data via CT scans whereas biophysical models depend on multiple tissue properties that are impractical to measure [11, 38]. We directly optimize the likelihood of the observed ablation zones on follow-up CT [29], the standard time point for treatment verification, therefore incorporating post-ablation processes such as tissue contraction and healing [39, 40]. Whereas biophysical models lack such feedback and only model thermal dose and ensuing cell death. Our algorithm is faster (inference takes a fraction of a second) making it suitable for clinical implementation [11].

There are several limitations of this work. Our dataset is small, which limits the ability of our current model to generalize to uncommon ablation zones. This can be overcome with more data. The model does not include intra-operative changes that likely account for multiple failed cases discussed before. The model also assumes the accurate position of the applicator, which can be difficult to ascertain from follow-up imaging in some cases. The model is trained to predict the ablation zone on the one-month follow-up scan, which correlates with necrotic tissue [41]. Moreover, the one-month follow-up scan has been shown to be a clinically relevant surrogate for necrosis in studies that correlate ablation zone margin with clinical outcomes [4, 39, 40] and hence is a clinically relevant endpoint for prediction. Notably, common sources of noise on early post-procedure imaging such as hemorrhage, edema, and pneumothorax resolve by the standard of care one-month follow-up scan. We exclude ablations with multiple burns or multiple applicators, which limits the generalizability of the model. However, given enough input data, we believe the model will be able to accommodate all these factors as well. Our model does not directly incorporate histology information and it is possible that histology may affect heat. We note however that histology has been shown to be encoded in CT data and therefore may be indirectly encoded in the current model [42]. Incorporating additional relevant, non-imaging input data may be considered in future work.

Our method has several important potential clinical applications [43, 44]. Pre-procedure planning with our model can be used to establish applicator trajectory, power, and duration. These could be tuned to optimize the margin with the goal of decreasing local recurrence and is safer and cost-effective compared to intra-operative biopsy for margin control [45]. Further studies are needed to establish margin assessment using our tool. The model can be used to identify “weak points” in the ablation that may require additional treatment. The rapidity of prediction allows for real-time clinical tuning by the operator. By pre-defining a minimum ablation margin around the nodule, our model can predict a set of “optimal solutions” from which the operator can select. Finally, our approach can be applied more broadly to other organs and other treatment modalities.

In conclusion, we have presented a patient-specific data-driven deep-learning approach to predict the post-procedure lung ablation zone. The work demonstrates a novel application of deep learning for a common yet unresolved clinical problem with improvement over the current standards and the best results to date to our knowledge. We anticipate generalizations of our approach to have broad applications to treatment planning and prediction in oncology.

## Supplementary information

Electronic Supplementary Material

## Abbreviations

ACCS: Applicator-centric co-ordinate system
CI: Confidence interval
CT: Computerized tomography
CV: Cross validation
HU: Hounsfield unit
IQR: Interquartile range
kVp: Kilovoltage peak
LLL: Left lower lobe
LOA: Limits of agreement
LUL: Left upper lobe
METS: Metastatic disease
MWA: Microwave lung ablation
RLL: Right lower lobe
RML: Right middle lobe
RUL: Right upper lobe
TRE: Target registration error
TT: Train-test
TV: Train-validation

## Footnotes

1 Study was determined as low risk; the patient consent requirement was waived.
