# Supplementary material for: Pre-operative lung ablation prediction using deep learning
Source: Eur Radiol. 2024 May 22;34(11):7161–72. doi: 10.1007/s00330-024-10767-8 (PMC11519138; doi:10.1007/s00330-024-10767-8)
Supplement: Supplementary file 1 — Electronic Supplementary Material [file 330_2024_10767_MOESM1_ESM.pdf]

Pre-operative lung ablation prediction using deep learning

<u>Electronic Supplementary Material (ESM)</u>

Eur Radiol (2024) Keshavamurthy KN, Eickhoff C, Ziv E

Supplementary material

A. Model

Our model was a fully convolutional neural network based on the U-Net [1] architecture (Figure 2d). The network consists of a total of 5 levels in the encoder/decoder, where the 5th level is the bottleneck of the U-Net. Starting with 32 filters in the first level, the number of filters is doubled at every successive level of the encoder until reaching the capped value of 320. The decoder follows a similar pattern where the number of filters is halved every successive level, resulting in 32 feature maps at the final decoder output. The input feature resolution is halved in successive encoder levels, whereas it is doubled in successive decoder levels.

Each level of the encoder/decoder is comprised of two blocks, where each block consists of a convolution followed by instance normalization [2] and leaky rectified linear unit (ReLU) non-linear [3] operations. Both blocks of an encoder level perform convolutions using 3x3x3 kernels, but use different strides except in the first level where they are the same. The first block uses a stride of 2x2x2 to simultaneously down-sample the input feature maps, whereas the second block uses a stride of 1x1x1 that maintains the feature map resolution as is. At each level of the decoder, the feature maps are first up-sampled using transpose convolution operations with a 2x2x2 kernel and stride 2x2x2. This is followed by two blocks, where each block performs a convolution using a 3x3x3 kernel with stride 1x1x1. In the first block, the convolution operation is performed on the up-sampled features concatenated with the features from the corresponding encoding layer. This doubles the number of feature channels intermittently, which is reduced to the original number of up-sampled feature channels by the following convolution operation with half the number of convolution filters. The second block performs the usual convolution, maintaining the size of the feature maps as is. The last layer of the network performs 1x1x1 convolutions using two filters to yield two feature maps (also called logits), one for the foreground class (ablation zone) and one for the background class (background lung parenchyma). Finally, a voxel-wise softmax function is applied to this output to obtain the final network prediction.

Deep supervision:

Deep supervision in deep networks is the method of introducing auxiliary supervision to the hidden layers in addition to the final output layer. Local outputs are added to the hidden layers,

allowing the gradients to flow from these as well as the final output layer during backpropagation [4]. This enables the gradients to be injected into deeper layers of the network, aiding in the training of all the layers and facilitating faster convergence [5]. In our network, deep supervision is added to all but the two lowest resolution decoder layers. The output feature maps of these layers are convolved with two 1x1x1 filters followed by voxel-wise softmax operation, just like in the final output layer of the network. The resulting map is compared with a corresponding down-sampled version of the ground truth segmentation mask and the resulting gradient is backpropagated into the hidden layer along with the final output gradient [1]. These additional outputs are only used during training and removed from the network during inference.

B. Training methodology

Nested cross validation:

(continued below...)

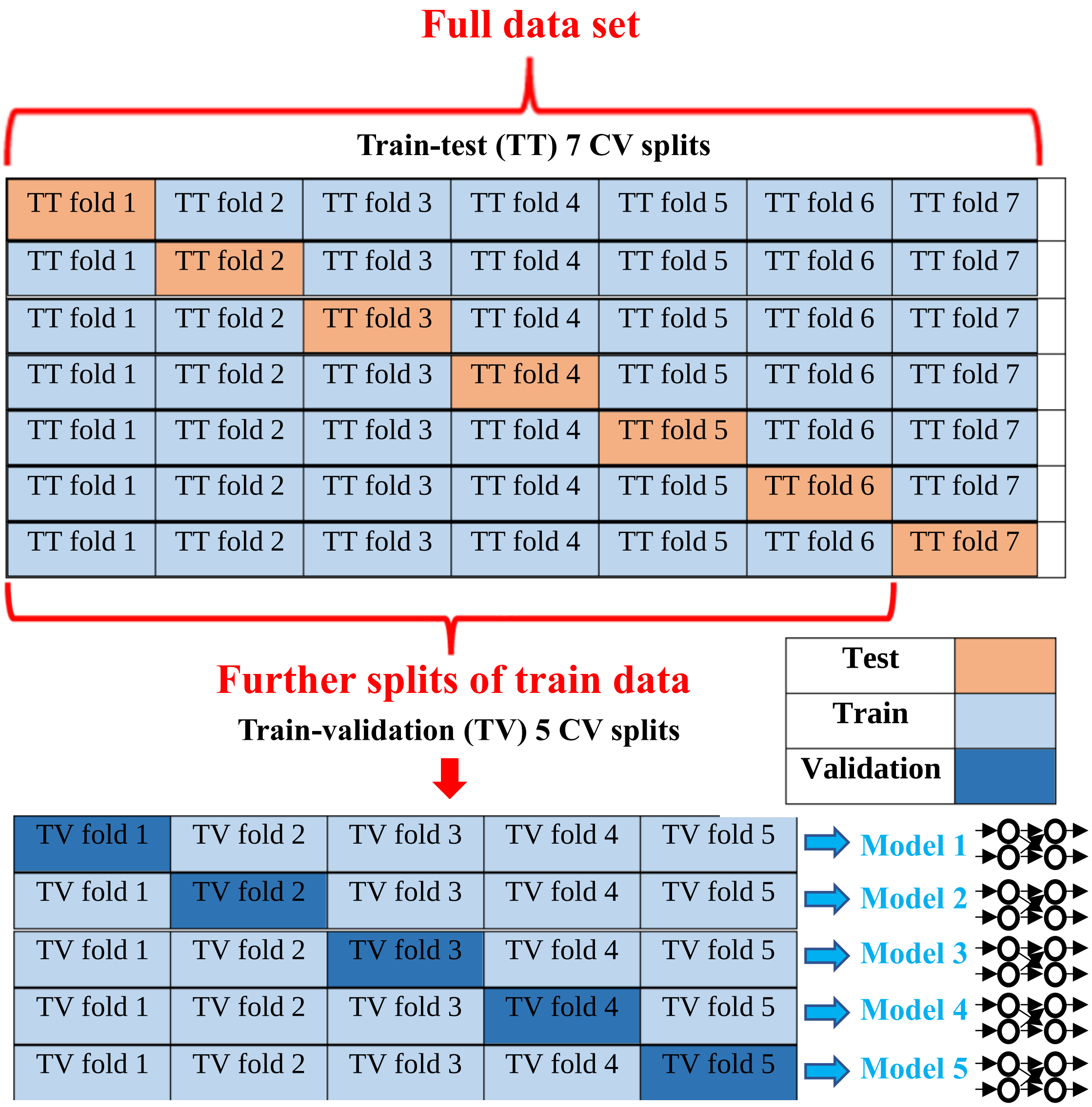

Figure B.1: Nested cross validation schema for training and testing. Top table: 7-fold cross validation (CV) on the full data, resulting in 7 train-test (TT) CV splits, represented by the different rows. Bottom table: Training data in each TT split further split into 5-fold train-validation (TV) splits, represented by the different rows. Algorithms were trained and tuned on each of these TV splits, one per split, resulting in 5 models from the training data of each TT split. These were applied on the corresponding test fold of the TT split unseen during training and the results averaged. This was repeated across all TT splits, resulting in a total of 7x5=35 trained models, and the results averaged across all the test folds of the TT splits to compute test set performance.

Training method:

We follow Isensee et al. [1] for the training methodology. All models were trained for a total of 1000 epochs, where each epoch consisted of 250 mini-batches randomly selected from the training set. The batch size was set to 19. The algorithm was trained using the stochastic gradient descent optimizer with Nesterov momentum (μ=0.99). The learning rate was initially set to value of 0.01 and was decayed using the "poly" learning rate schedule [6] (which is similar to a linear decrease to 0) during the course of training. For deep supervision, the total loss was computed as the weighted sum of the individual losses at each resolution (levels of the decoder). The weights were halved in successively decreasing resolutions and were normalized such that they summed to 1. The data was augmented during training using rotations, translations, scaling, mirroring, addition of Gaussian noise/blur, variations in brightness, contrast, simulation of low resolution etc. The CT scan intensities were normalized by first cropping them to 0.5 and 99.5 percentile values across all images and subtracting the mean and dividing by the standard deviation computed from all images. The binary vendor model (1s inside the vendor estimated ablation zone and 0s outside) was included as an additional channel along with the CT scan as input to the network. During inference, test data was augmented by mirroring along all axes. The nnU-Net Python framework [1] was used for model implementation. All training was performed on an Nvidia Quadro RTX 8000 GPU with 48 GB of RAM and took approximately 8 hours per session.

C. Target registration error (TRE)

The average Euclidean distance between corresponding anatomical landmarks in the two images after registration [7, 8].

I.e., $TRE = \sum_i \| T(p_f^i) - p_m^i \|$,

where T is the registration transformation, p_f^i and p_m^i are the ith points in the fixed and moving images, respectively, that are known to correspond, and $\|.\|$ is the Euclidean distance. Note that the points p_f^i and p_m^i should not have been used to estimate the registration transform T.

D. Dice, precision, recall

Let P, G be prediction and ground truth 3D objects, and P_{vol} and G_{vol} be their volumes, respectively. Then,

1) Dice score [9]**:**

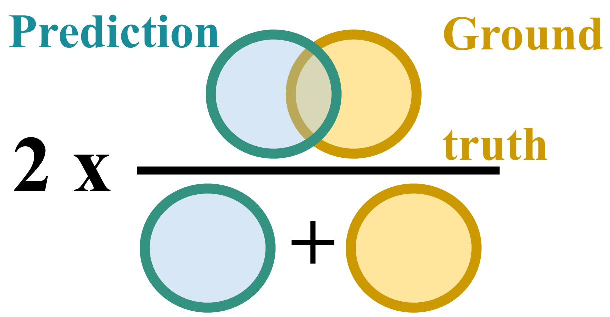

i.e., Dice = 2 × $\frac{P \cap G}{P_{vol} + G_{vol}}$**.**

2) Precision [10]**:**

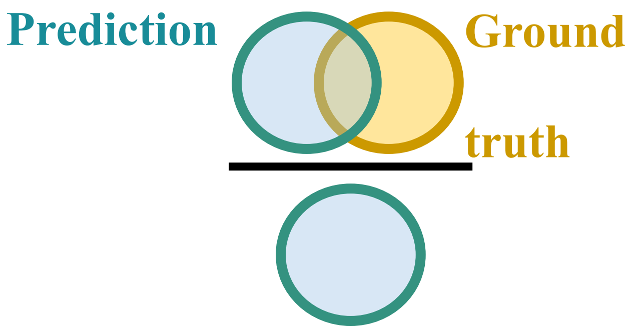

i.e., Precision = $\frac{P \cap G}{P_{vol}}$**.**

3) Recall [10]

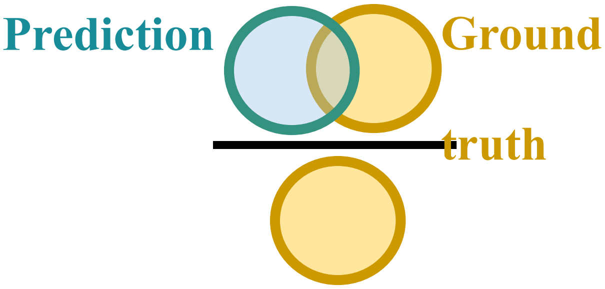

$$\text{i.e., Recall} = \frac{P \cap G}{G_{vol}}.$$

E. **Improvement in Dice score is an under-estimate**

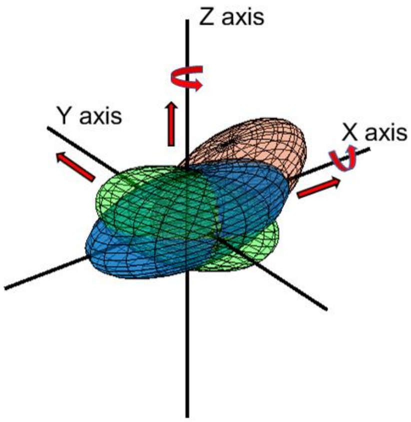

Figure E.1: Effect of misregistration on Dice score. A reference ellipsoid is placed at the origin with major axis lying along the x axis (shown in blue). New ellipsoids are created (e.g., green, orange) that are translated and rotated (shown by red arrows) and their Dice scores with the reference computed.

The added step of registration for our model introduces a bias against our model relative to the vendor model, which doesn't need to undergo registration. This is because, all the computations are performed in the followup post scan space. The applicator position is extracted on the followup scan and the vendor model is placed at that position, and hence is already in the followup post scan space and perfectly registered. Whereas, the prediction is performed on the

pre scan, which is registered to the followup post scan to bring it over to the followup post scan space, subjecting it to mis-registration and hence lower Dice scores.

To quantify the effect of mis-registration on Dice score, we computed Dice scores between ellipsoids of similar dimensions but at different relative positions and orientations. The ellipsoid dimensions were chosen such that they corresponded with the vendor ablation zone at 65 W and 5 min, as these were the median ablation power and duration settings in our data set. Figure E.1 illustrates the procedure. The reference ellipsoid was placed at the origin, with the major axis oriented along the x axis. New ellipsoids were then created by perturbing the reference ellipsoid via translations along different directions as well as rotations about different axis. All rotations were first applied at the origin followed by translations to different locations. The translations varied from 0-5 mm and the rotations varied between 0°-45°. Both in-plane and out-of-plane transformations were performed. Dice scores were then computed in each of these scenarios between the perturbed and the reference ellipsoid as shown in Table E.1. We saw large effects on the Dice score (10-20% decrease) even with small perturbations in translation (2 mm) or angle of rotation (15°). Notably, unlike our models, the vendor model is applied directly to the followup post scan (from which the applicator coordinates are extracted) and does not involve any registration and is therefore not subject to this registration effect. Thus, the true improvement over the vendor model in the Dice score is likely higher.

Table E.1: Impact of misregistration on Dice score. Listed are Dice scores between ellipsoids that are misregistered by 0-5 mm in position and by 0 to 45° in orientation.

**In-plane rotations and translations**				
**Angle of rotation about z axis**	**No translation**	**2 mm translation along x axis**	**2 mm translation along y axis**	**2 mm translation along x=y line in-plane**
0°	1.0	0.91	0.84	0.87
15°	0.88	0.87	0.83	0.87
45°	0.71	0.70	0.70	0.70
**Angle of rotation about z axis**	**No translation**	**3 mm translation along x axis**	**3 mm translation along y axis**	**3 mm translation along x=y line in-plane**
0°	1.0	0.87	0.76	0.81
15°	0.88	0.84	0.76	0.85

45°	0.71	0.69	0.68	0.69
**Angle of rotation about z axis**	**No translation**	**5 mm translation along x axis**	**5 mm translation along y axis**	**5 mm translation along x=y line in-plane**
0°	1.0	0.79	0.61	0.69
15°	0.88	0.77	0.62	0.77
45°	0.71	0.65	0.62	0.65
**Out-of-plane rotations and translation**				
**Angle of rotation about z axis and angle of rotation from x-y plane in series**	**No translation**	**2 mm translation along oriented direction in 3D**	**3 mm translation along oriented direction in 3D**	**5 mm translation along oriented direction in 3D**
15°	0.84	0.83	0.81	0.75
45°	0.65	0.64	0.64	0.60

F. Trade-off between precision and recall

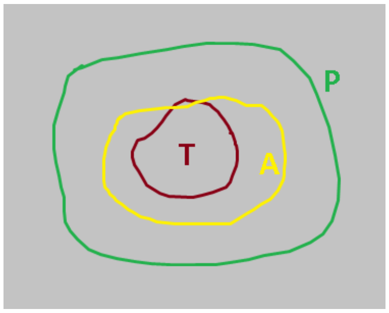

High recall; low precision

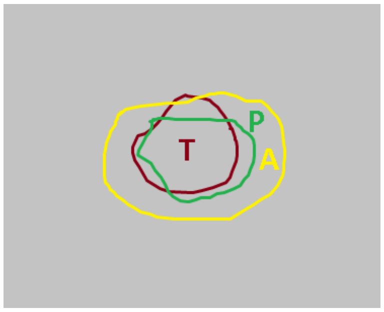

Low recall; high precision

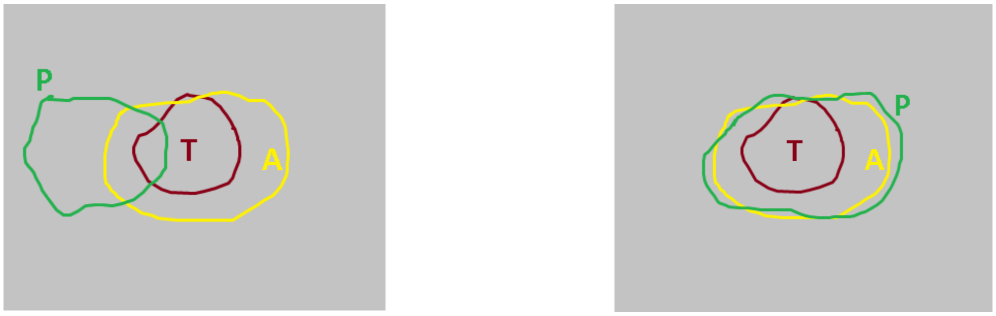

Low recall; low precision **High recall; high precision**

Figure F.1: Illustrations of various precision-recall scenarios of the predicted ablation zone (shown in green and denoted by P) with respect to the actual ablation zone (shown in yellow and denoted by A). The tumor is shown in maroon and is denoted by T.

G. More details on our novel registration methodology optimized for ablation workflows.

Our method is based on a two-stage process: 1) in the first stage, we perform an initial rigid registration that ensures the images are globally aligned and provides a good initialization for the subsequent deformable registration; and 2) in the second stage, we perform multi-resolution deformable image registration to account for the non-rigid motion of the lung anatomy to obtain a finer match [11, 12]. We additionally incorporate various constraints optimized for ablation workflows that aid in achieving successful registrations. Below we describe the various parameters of the registration.

Parameters used for registration:

1) *Focusing on the region of interest (ROI):* We are only interested in registering the anatomy close to the regions of interest, i.e., tumor in pre scan and ablation zone in follow-up post scan. The ablation prediction is not affected by anatomical features far away. E.g., the ablation zone in the left lung is not directly influenced by the blood vessels in the right lung. We achieve this by **first 1) cropping** the images in 3D using spherical masks of chosen radii (e.g., 70mm), centered at the segmentations. This results in spherical images with the tumor/ablation zone at the center, removing any effect of far-away structures as well as speeding up registrations since the cropped images are smaller; and **more importantly using 2) registration masks** to constrain the registration to be driven by only the intensities inside a further smaller spherical region centered on the tumor/ablation zone (e.g., 30-50mm radius). These ensures the immediately surrounding anatomy of tumor/ablation zone has the most impact on the registration, increasing the likelihood of accurate registrations in these regions.

We remove the tumor and the ablation zone segmentations themselves from these spherical masks to ensure they do not contribute to the registration process. This is done to avoid the non-rigid transform from attempting to match the tumor with the bigger ablation zone by expanding/altering its size and shape.

2) *Image similarity metric:* image mutual information [13]; the number of intensity histogram bins for computing the joint distribution was chosen to be 32.
3) *Optimizer:* stochastic gradient descent, where "step size" was adapted as a function of the similarity between the gradient directions in the current and the previous iterations [14]. The maximum number of iterations was set to 256.
4) *Image sampler:* image intensities were sampled from 2048 locations (including sub-voxel) from the image regions inside the specified registration masks, with new spatial samples selected in every iteration.
5) *Image interpolation:* We used tri-linear interpolation during the registration. For computing the final transformed image, B-spline based cubic interpolation is used for better quality.
6) *Initial rigid registration:* The images are first aligned using a simpler rigid transformation before proceeding to the more complex non-rigid deformations. This ensures the images are globally aligned first and provides a good initialization for the subsequent non-rigid registrations [11, 12]. The rigid transformation was composed of a 3D rotation matrix and a translation vector. The centroids of tumor in pre and ablation zone in follow-up post are also matched with a certain weight in the loss function to guide the initial alignment.
7) *Non-rigid/deformable registration:* We utilize the free form deformation model (FFD) based on B-splines [15] as the transformation function. FFDs are defined by a grid of control points which can be used to manipulate the shape of 3D objects. Using cubic B-splines, a hyperpatch is defined by the control points in 3D that is smooth and continuous. Moving the control points of the FFD deforms the B-spline transformation function, which in turn results in smooth deformations of the underlying fixed image pixel grid. The parameters of the transform are the positions of the control points, which are optimized during the registration to maximize the similarity between the deformed moving and fixed images.
8) *Regularization:* We utilized the bending energy as our regularizer [15, 16], which penalizes abrupt variations in the transformation (e.g., high expansion followed by high compression) and can avoid folding.
9) *Multi-resolution strategies:* A coarse-to-fine hierarchical image registration at multiple resolutions was performed, where images were smoothed using specified Gaussian kernels to different resolutions and registered incrementally starting with the lowest [12]. The lowest

resolutions reduce the number of local optima, increasing the likelihood of finding the global optima. This strategy aligns large structures first, with the finer details introduced and matched at successively higher resolutions. The number of resolutions was chosen to be 4, with the registration proceeding from the lower to the higher resolutions in succession. The lower resolution images were obtained by smoothing the image with a Gaussian of $\sigma = 0.5$ in the finest resolution and increased by a factor of 2 in each successive lower resolution. A similar strategy is followed for the B-spline transformation, where the complexity of the transform (i.e., the number of individual parameters in the transform) is increased from low to high during successive registrations at increasingly higher resolution levels [11, 15]. At lower image resolutions, a coarse B-spline control point grid is used with higher spacing between control points; this allows for only coarse deformations, aiding to match larger global structures. Whereas at higher resolutions, a finer grid is used with lesser spacing between the control points; this allows for local deformations, aiding to match smaller local structures in the images. The control point grid spacing started with 8 mm along all dimensions at the finest resolution and was increased to 11.28, 15.84, and 22.4 mm in successive lower resolutions.

10) *Rigidity penalty on tumor region:* While non-rigid registration is necessary to match the anatomy surrounding the tumor in the pre and ablation zone in the followup post scans, we desire the tumor area itself to not undergo large deformations. Preserving volume and shape morphology of the tumor is necessary to learn a meaningful relationship with the ablation zone. We achieve this by imposing a rigidity penalty on the tumor region in the pre scan following Staring et al. [17]. The specific constraints include: **1) Affinity condition:** This condition ensures that the second order partial derivatives of the transform with respect to the spatial coordinates is 0, as required by a rigid transform (and more generally an affine transform); **2) Orthonormality condition:** This condition enforces the property that the rotation matrix should be orthonormal; and **3) Properness:** This condition ensures that the determinant of the rotation matrix is 1. This is equivalent to imposing the determinant of the Jacobian of the transform is 1, which implies no change in volume (incompressibility [18]). The final rigidity penalty is a weighted combination of the above conditions for e.g., in the ratio 100:1:10. The region where this needs to be imposed, the tumor region in our case, is specified via a 0, 1 tumor segmentation mask. For B-spline s transformations, the rigidity penalty is imposed via the control points, taking advantage of the local support of B-splines. E.g., to ensure rigidity at a point x, all the control points in the local support of the B-spline

that influence x are forced to be rigid. Rigidity of the control points ensures rigidity of the object.

11) *Overall registration optimization cost function:* The overall registration optimization cost function included the following terms: similarity metric, the bending energy regularization, the rigidity penalty, and the ROI (tumor/ablation) centroid matching term for rigid registration, all of whose weights were set to 1.

All registrations were performed using the SimpleElastix image registration library [11, 19]. We set several of the registration parameters above following Klein et al. [11].

H. Example 3D figures of our predicted ablation zone, true ablation zone and vendor model

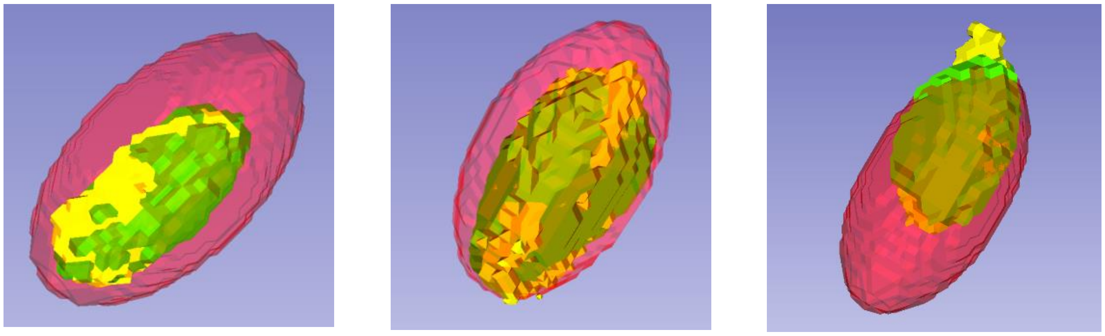

(a) **(b)** **(c)**

Figure H.1: 3D examples of our predicted ablation zone (green), true ablation zone (yellow) and vendor predicted ablation zone (red). Notice that in each of the 3 cases, our prediction closely follows the true ablation zone, whereas the vendor significantly overestimates it.

Bibliography

1. Isensee F, Jaeger PF, Kohl SAA, et al (2021) nnU-Net: a self-configuring method for deep learning-based biomedical image segmentation. Nat Methods 18:203–211

2. Ulyanov D, Vedaldi A, Lempitsky V (2016) Instance normalization: The missing ingredient for fast stylization. arXiv preprint arXiv:160708022

3. Maas AL, Hannun AY, Ng AY (2013) Rectifier nonlinearities improve neural network acoustic models. In: Proc. icml. Atlanta, Georgia, USA, p 3

4. Lee C-Y, Xie S, Gallagher P, et al (2015) Deeply-supervised nets. In: Artificial intelligence and statistics. PMLR, pp 562–570

5. Turečková A, Tureček T, Komínková Oplatková Z, Rodríguez-Sánchez A (2020) Improving CT image tumor segmentation through deep supervision and attentional gates. Front Robot AI 7:106

6. Chen L-C, Papandreou G, Kokkinos I, et al (2017) Deeplab: Semantic image segmentation with deep convolutional nets, atrous convolution, and fully connected crfs. IEEE Trans Pattern Anal Mach Intell 40:834–848

7. Datteri RD, Dawant BM (2012) Estimation and reduction of target registration error. In: Medical Image Computing and Computer-Assisted Intervention–MICCAI 2012: 15th International Conference, Nice, France, October 1-5, 2012, Proceedings, Part III 15. pp 139–146

8. Insight Software Consortium (2023) Registration Errors, Terminology and Interpretation. In: Accessed on. https://insightsoftwareconsortium.github.io/SimpleITK-Notebooks/Python_html/68_Registration_Errors.html. Accessed 13 Nov 2023

9. Dice LR (1945) Measures of the amount of ecologic association between species. Ecology 26:297–302

10. Powers DMW (2020) Evaluation: from precision, recall and F-measure to ROC, informedness, markedness and correlation. arXiv preprint arXiv:201016061

11. Klein S, Staring M, Murphy K, et al (2009) Elastix: a toolbox for intensity-based medical image registration. IEEE Trans Med Imaging 29:196–205

12. Lester H, Arridge SR (1999) A survey of hierarchical non-linear medical image registration. Pattern Recognit 32:129–149

13. Viola P, Wells III WM (1997) Alignment by maximization of mutual information. Int J Comput Vis 24:137–154

14. Klein S, Pluim JPW, Staring M, Viergever MA (2009) Adaptive stochastic gradient descent optimisation for image registration. Int J Comput Vis 81:227–239

15. Rueckert D, Sonoda LI, Hayes C, et al (1999) Nonrigid registration using free-form deformations: application to breast MR images. IEEE Trans Med Imaging 18:712–721

16. Wahba G (1990) Spline models for observational data. SIAM

17. Staring M, Klein S, Pluim JPW (2007) A rigidity penalty term for nonrigid registration. Med Phys 34:4098–4108

18. Rohlfing T, Maurer CR, Bluemke DA, Jacobs MA (2003) Volume-preserving nonrigid registration of MR breast images using free-form deformation with an incompressibility constraint. IEEE Trans Med Imaging 22:730–741

19. Marstal K, Berendsen F, Staring M, Klein S (2016) SimpleElastix: A user-friendly, multi-lingual library for medical image registration. In: Proceedings of the IEEE conference on computer vision and pattern recognition workshops. pp 134–142
